# Towards the validation of high-throughput sequencing (HTS) for routine plant virus diagnostics: measurement of variation linked to HTS detection of citrus viruses and viroids

**DOI:** 10.1186/s12985-021-01523-1

**Published:** 2021-03-22

**Authors:** Rachelle Bester, Glynnis Cook, Johannes H. J. Breytenbach, Chanel Steyn, Rochelle De Bruyn, Hans J. Maree

**Affiliations:** 1 Department of Genetics, Stellenbosch University, Private Bag X1, Matieland, 7602 South Africa; 2 Citrus Research International, P.O. Box 28, Nelspruit, 1200 South Africa; 3 Department of Plant Pathology, Stellenbosch University, Private Bag X1, Matieland, 7602 South Africa; 4 Citrus Research International, P.O. Box 2201, Matieland, 7602 South Africa

**Keywords:** CTV, Citrus tristeza virus, Next-generation sequencing, Bioinformatics

## Abstract

**Background:**

High-throughput sequencing (HTS) has been applied successfully for virus and viroid discovery in many agricultural crops leading to the current drive to apply this technology in routine pathogen detection. The validation of HTS-based pathogen detection is therefore paramount.

**Methods:**

Plant infections were established by graft inoculating a suite of viruses and viroids from established sources for further study. Four plants (one healthy plant and three infected) were sampled in triplicate and total RNA was extracted using two different methods (CTAB extraction protocol and the Zymo Research Quick-RNA Plant Miniprep Kit) and sent for Illumina HTS. One replicate sample of each plant for each RNA extraction method was also sent for HTS on an Ion Torrent platform. The data were evaluated for biological and technical variation focussing on RNA extraction method, platform used and bioinformatic analysis.

**Results:**

The study evaluated the influence of different HTS protocols on the sensitivity, specificity and repeatability of HTS as a detection tool. Both extraction methods and sequencing platforms resulted in significant differences between the data sets. Using a de novo assembly approach, complemented with read mapping, the Illumina data allowed a greater proportion of the expected pathogen scaffolds to be inferred, and an accurate virome profile was constructed. The complete virome profile was also constructed using the Ion Torrent data but analyses showed that more sequencing depth is required to be comparative to the Illumina protocol and produce consistent results. The CTAB extraction protocol lowered the proportion of viroid sequences recovered with HTS, and the Zymo Research kit resulted in more variation in the read counts obtained per pathogen sequence. The expression profiles of reference genes were also investigated to assess the suitability of these genes as internal controls to allow for the comparison between samples across different protocols.

**Conclusions:**

This study highlights the need to measure the level of variation that can arise from the different variables of an HTS protocol, from sample preparation to data analysis. HTS is more comprehensive than any assay previously used, but with the necessary validations and standard operating procedures, the implementation of HTS as part of routine pathogen screening practices is possible.

**Supplementary Information:**

The online version contains supplementary material available at 10.1186/s12985-021-01523-1.

## Background

The prevention and management of plant diseases largely depend on accurate identification of pathogens. Rapid and specific detection assays are required that need continuous development and optimisation as technology and knowledge of the pathogens advance. The increased use of high-throughput sequencing (HTS) for the construction of virome profiles of many agricultural crops has led to the discovery of many new viruses [1–13]. Some viruses cause, or are associated with, economically damaging diseases necessitating detection tools that can reliably detect them in the shortest timeframe. The successful control of viruses and viroids in commercial crops are directedly correlated to the effectiveness of the detection assays used to screen plant propagation material. The challenges and opportunities of HTS for virus and viroid detection has been highlighted previously [14–17] and in the United States of America, HTS already forms part of their clean plant propagation programs by creating a provisional release category based on a HTS-negative selection [16]. This category of plants is then allowed to be propagated in designated approved areas pending the completion of all conventional laboratory tests. This allows accelerated multiplication of plant material prior to official clean status certification and release.

The generation of sequence data using various metagenomic and enrichment strategies and the development of the associated bioinformatic pipelines has led to the identification of many known and novel viral sequences [18]. The confirmation of these agents is usually subsequently verified using a PCR or RT-PCR assay designed using the HTS data generated in the first place. The question is therefore whether HTS can be used as a reliable standalone detection tool if the necessary parameters i.e. sensitivity and reliability can be validated as for any other detection assay. As the use of HTS is more routinely employed, the need to establish the influence of variables such as sample preparation, sequencing platforms and data analysis on the output becomes imperative. If HTS can be validated for sensitivity and specificity within a specific pathosystem, it can be used as a standalone detection assay to provide a fast and reliable diagnostic for any viral disease. For known viruses, a HTS detection assay has great application value for broad based detection of viruses in high value plant material at the post import quarantine stage or in the clean-status verification of nuclear or mother plant material of plant improvement schemes. One limitation to this assay would be the comprehensiveness of the reference database used.

The current consensus is that HTS data analyses and the interpretation of the results for plant viral diseases require expertise in both bioinformatics and plant virology [14, 15]. Nevertheless, numerous studies report attempts to streamline the analysis and to find a one size fits all solution to the bioinformatic component of the HTS assay to inform a diagnostic call [19–36]. There are also different options for target nucleic acids to be sequenced, most popular being total RNA (commonly ribo-depleted), small RNA (sRNA) and double stranded RNA depending on the application of the HTS assay. A popular strategy employed for the detection of plant viruses is sRNA sequencing. This strategy can effectively detect viruses with DNA or RNA genomes. However, sRNAs are generated as a host defence response to virus infection and a weaker response will result in lower levels of sRNAs that could impact negatively on this approach’s ability to detect these specific viruses [37, 38]. The effect of different bioinformatic pipelines was also evaluated previously in a large-scale performance test on sRNA data and the variation in results were significant [39]. The advantages of ribo-depleted RNA sequencing over sRNA sequencing for virus detection were also highlighted previously [37, 40].

Even though plant virologists without training in bioinformatics can benefit from automated pipelines with graphical user interfaces, the quality and accuracy of the output is reliant on the quality of the input. The input to an HTS assay incorporates the whole process from sample collection, wet laboratory processing and data generation including data quality control. All the quality control measures up to data QC are the same or similar to any other sensitive molecular assay and need to be incorporated as assay variables. To ensure optimum data analysis, data should be evaluated for the different quality parameters, including not only the quality scores of each base, but also the sequencing depth. All these parameters can impact on the specificity, sensitivity and repeatability of the diagnostic result. The specific application of HTS will determine the acceptable level of variation that is tolerable. Identifying the exact virus or viroid variant, for example, is not required for pathogen detection. Applications of HTS are therefore varied and include both detection and discovery. It is important to identify the application and to tailor the assay, data analysis and interpretation accordingly.

The citrus industry is one of the largest fruit industries worldwide with South Africa being the second largest fresh citrus exporter [41]. However, citrus pathogens can lead to a reduction in yield and threaten cultivar sustainability. One of the most devastating and complex viral pathogens of citrus species locally and worldwide is the closterovirus, citrus tristeza virus (CTV) [42, 43]. Other pathogens sporadically detected in non-certified citrus in South Africa include citrus tatter leaf virus (CTLV) and viroids such as hop stunt viroid (HSVd), citrus dwarfing viroid (CDVd) and citrus exocortis viroid (CEVd). More recently citrus virus A (CiVA) was detected in older orchards [44].

Viruses and viroids that are mainly spread through vegetative propagation can be effectively controlled through the use of certification programmes to generate virus free budwood/cuttings for propagation. HTS can detect multiple pathogens within a single assay, and has the advantage that data can be re-evaluated as new viral agents and variants are added to global databases.

In this study, *Citrus sinensis* plant material infected with a range of viruses, including positive and negative sense RNA viruses, and viroids were established and subjected to HTS to evaluate the level of biological and technical variation that can arise from the RNA extraction method, sequencing platform and bioinformatic pipeline used. This study evaluated HTS variation for a citrus virome in order to use HTS as a standalone detection assay.

## Methods

### Plant material

**Fig. 1:**
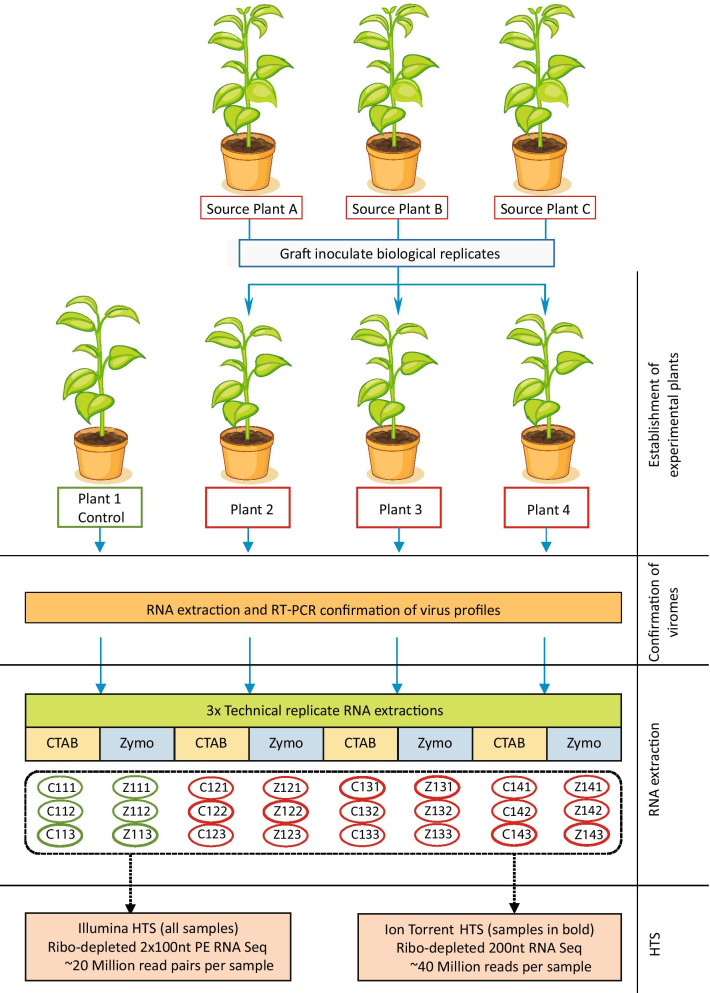
Visual representation of experimental layout. The establishment of plant material and the selection of samples subjected to high-throughput sequencing (HTS) is illustrated. Source plant A is infected with citrus tristeza virus (CTV) (genotypes RB, T3, T30, VT and S1), citrus virus A (CiVA), hop stunt viroid (HSVd), citrus dwarfing viroid (CDVd) and citrus exocortis viroid (CEVd), source plant B with only ‘*Candidatus* Liberibacter africanus’ (CLaf)) and source plant C with citrus tatter leaf virus (CTLV)

A set of plants (*C. sinensis* cv. ‘Madam Vinous’, sweet orange) were established from seed and each graft inoculated in February 2019 with three source plants infected with a pre-determined range of pathogens (citrus tristeza virus (CTV), citrus virus A (CiVA), citrus tatter leaf virus (CTLV), hop stunt viroid (HSVd), citrus dwarfing viroid (CDVd) and citrus exocortis viroid (CEVd)). Source plant A was infected with CTV genotypes RB, VT, T3, T30, S1 and A18, CiVA, HSVd, CDVd and CEVd, source plant B with ‘*Candidatus* Liberibacter africanus’ (CLaf) and source plant C only contained CTLV. These source plants are well characterised sources maintained at Citrus Research International (CRI) in Nelspruit, South Africa. Plants were maintained in a temperature-controlled greenhouse (24–28 °C) with natural light in summer, but with additional lighting provided in winter months to supply a total of 16 h light per day. The infection status of the graft inoculated plants were confirmed with RT-PCR eight months post inoculation. Briefly, total RNA was extracted from leaves using an acid phenol extraction buffer [45]. Complementary DNA (cDNA) was synthesized from 1 μg of total RNA using 0.1 μg of random hexamers (Inqaba Biotec), dNTPs (1 mM final concentration) (Thermo Scientific), 100 U of RevertAid H Minus Reverse Transcriptase (Thermo Scientific) and 10 U of RiboLock RNase Inhibitor (Thermo Scientific) in a final volume of 20 μl according to manufacturer’s instructions. A 1 μl aliquot of cDNA was added to 9 μl of PCR reaction mixture containing 1 × GoTaq® G2 Hot Start Master Mix (Promega) and 0.375 μM forward and reverse primers (IDT) (Additional file 1). Cycle conditions for the different assays included an initial denaturation step at 94 °C for 2 min, followed by 35 cycles of 94 °C for 20 s, primer specific annealing temperature (Additional file 1) for 60 s, elongation at 72 °C for 20 s and a final extension of 72 °C for 5 min. One healthy plant and three plants infected with a pathogen complement including HSVd, CDVd, CEVd, CTV, CiVA and CTLV were selected for further analysis (Fig. 1).

### Total RNA extractions for HTS

A representative leaf sample was taken from each of the four experimental plants, and from this sample three random samples were taken for further processing. Total RNA was extracted from these three samples per plant using two different extraction methods (Fig. 1). One gram of leaf midribs of each sample were homogenised in a Bioreba extraction bag and total RNA extracted using a modified CTAB extraction protocol [46]. An *Ornithogalum thyrsoides* leaf sample infected with Ornithogalum mosaic virus (OrMV) was also subjected to the CTAB extraction protocol as a nontarget positive control. The Quick-RNA Plant Miniprep Kit (Zymo Research) was used to extract total RNA from 0.2 μg of leaf midribs from each sample homogenised in liquid nitrogen. The three samples from each of the four plants were sequenced on the Illumina HTS platform and one sample of each plant was sequenced on the Ion Torrent HTS platform. For the samples sequenced on both platforms, the same RNA extract was divided and shipped to the respective service providers. The quality of the RNA was assessed independently at each service provider using the Agilent 2100 BioAnalyzer to determine the RNA integrity number (RIN).

### CTV RT-PCR genotyping

Two-step RT-PCRs were performed to determine the CTV genotype status of the graft inoculated plants. Complementary DNA (cDNA) was synthesized from 1 μg of total RNA using 0.15 μg of random hexamers (Promega) and Maxima reverse transcriptase (Thermo Scientific) in a final volume of 20 μl according to manufacturer’s instructions. A 2-μl aliquot of cDNA was added to 25 μl of PCR reaction mixture containing 1 × KAPA Taq buffer A (Roche), 0.2 mM dNTP mix (Thermo Scientific), 0.4 μM forward and reverse primers (IDT) (Additional file 1), and 1.25 U/μl KAPA Taq DNA polymerase (Roche). Cycle conditions for the different assays included an initial denaturation step at 94 °C for 5 min, followed by 35 cycles of 94 °C for 30 s, primer specific annealing temperature (Additional file 1) for 30 s, and elongation at 72 °C for 30 s and a final extension of 72 °C for 7 min.

### High-throughput sequencing

Twenty-four ribo-depleted RNA libraries, representing three technical replicates of RNA extracted from four *C. sinensis* plants with two total RNA extraction methods, were constructed with the Illumina TruSeq Stranded Total RNA Sample Preparation kit with Plant Ribo-Zero at Macrogen (South Korea) (Fig. 1). Paired-end HTS (2 × 100 bp) was performed on an Illumina NovaSeq 6000 instrument (Macrogen, South Korea). The nontarget positive control RNA originating from *Ornithogalum* was also subjected to the Illumina sequencing protocol.

Eight RNA samples, representing one technical replicate of each of the four *C. sinensis* plants extracted with two RNA extraction methods, were ribo-depleted (RiboMinus™ Plant Kit for RNA-Seq, ThermoFisher Scientific), and sequencing libraries were constructed (Ion Total RNA-Seq Kit v2.0, ThermoFisher Scientific) (Central Analytical Facility (CAF), Stellenbosch University) (Fig. 1). Single-end (200 bp chemistry) high-throughput sequencing was performed on an Ion Torrent™ Proton™ instrument (CAF, Stellenbosch University).

The service providers were requested to provide 20 million read pairs per sample from Illumina sequencing and 40 million single end reads from Ion Torrent.

### Quality assessment of HTS data

Adapter sequences were removed from the Illumina data and data was trimmed for quality using Trimmomatic [47] (SLIDINGWINDOW of 3 nts with Q20, MINLEN of 20 nts). The Ion Torrent data was processed by CAF using the default pipeline of the Torrent Suite software V5.12.0 (Thermo Fisher) including the removal of polyclonal reads and low quality reads, 3′ trimming using a 30 nt moving window and a quality score of 15, 6 nt adapter match trimming and a removal of reads shorter than 8 nts.

### De novo assemblies

Both the trimmed Illumina and Ion Torrent data were subjected to de novo assemblies using SPAdes 3.14 [48] and CLC genomics Workbench 11.0.1 (CGW) (Qiagen) (default parameters). SPAdes de novo assembled scaffolds were identified using BLAST + standalone against a local copy of the NCBI GenBank nucleotide database using the Blastn algorithm. The scaffolds with no Blastn hits were mapped to the *C. sinensis* host genome (C.sinensis_Hzau_v2.0_genome) [49] and the unmapped scaffolds (classified as “Number of scaffolds not mapped” in Additional file 2) were subjected to Blastx against a local copy of the NCBI GenBank non-redundant database.

To perform the CGW assembly both the Illumina and Ion Torrent data were imported using the appropriate tool for Illumina or Ion Torrent fastq files, respectively. Subsequently the data was de novo assembled (default parameters) and the resulting contigs were identified using Blastn.

### Read mapping

All the quality trimmed reads were mapped to reference genomes extracted from Genbank. These references included the expected viruses and viroids (CiVA, MT720885, MT720886; CTLV, MH108976; CTV isolate B390-5, KU883265; CDVd, KY110718; CEVd, KY110721; HSVd, KY110716) as well as nontarget variants of expected viruses (CTV isolate GFMS12-8, MK033511; HSVd, KY110717), and the nontarget positive control virus (OrMV, KY769694.1). The Burrows-Wheeler Alignment Tool (BWA) version 0.7.13 [50] were utilised and the mapped reads filtered for 95% similarity for a 90% read fraction using samtools version 1.10 [51]. The genome coverage (span from the first to the last base of the genome) for each reference genome was calculated using samtools.

The CTV genotype status of each plant sample was also confirmed with read mapping using the pipeline and criteria established previously [52].

Additionally, 12 genes previously evaluated as *C. sinensis* reference genes were identified from literature [53] and retrieved from GenBank using the best Blastn hit (online NCBI tool) of the *Arabidopsis Thaliana* orthologs against the organism *C. sinensis* (Table 1). All the quality trimmed reads were mapped to the reference genes using BWA and the gene coverage (span from the first to the last base of the genome) for each reference gene was calculated using samtools.

**Table 1:** Gene and pathogen accessions used as reference sequences for read mapping

Accession			Name		Length
*Arabidopsis thaliana* Gene locus identifier	*Citrus sinensis* accession	Gene symbol	*Arabidopsis thaliana* gene name	*Citrus sinensis* gene name	
At2g28390	XM_006488024.3	SAND	SAND family protein	Vacuolar fusion protein MON1 homolog (LOC102625488)	2415
At5g08290	XM_006484464.3	DIM1	DIM1 homolog/YLS8	Thioredoxin-like protein YLS8 (LOC102629695)	727
At2g32170	XM_006481276.3	N/A	Unknown protein	Carnosine N-methyltransferase-like (LOC102617870)	1773
At5g15710	XM_006482390.2	FBOX	F-box family protein	F-box/kelch-repeat protein At5g15710 (LOC102621205)	4479
At3g53090	XM_025099888.1	UPL7	Ubiquitin-protein ligase 7	E3 ubiquitin-protein ligase UPL7 (LOC102621690)	3888
At5g25760	XM_006476013.2	UBC21	Ubiquitin-conjugating enzyme 21	Protein PEROXIN-4 (LOC102618324)	1029
At3g01150	XM_025099846.1	PTB1	Polypyrimidine tract-binding protein 1	Polypyrimidine tract-binding protein homolog 1 (LOC102618721)	1708
At1g13440	XM_006476919.3	GAPC2	Glyceraldehyde-3-phosphate dehydrogenase C2	Glyceraldehyde-3-phosphate dehydrogenase GAPC1, cytosolic (LOC102624117)	1464
At4g27960	XM_006490521.3	UBC9	Ubiquitin conjugating enzyme 9	Ubiquitin-conjugating enzyme E2 10 (LOC102614401)	961
At3g18780	XM_006464503.3	ACT2	Actin-2	Actin-7 (LOC102577980)	1786
At5g60390	XM_006485840.3	EF-1a	Elongation factor 1-alpha	Elongation factor 1-alpha (LOC102613486)	1870
At1G20010	XM_006473602.3	TUB	Beta-Tubulin	Tubulin beta-6 chain (LOC102631140)	1756
Genbank accession		Pathogen abbreviation	Pathogen name		
KU883265		CTV	Citrus tristeza virus B390-5 RB		19,270
MK033511		CTV	Citrus tristeza virus GFMS12-8 T68		19,246
MH108976.1		CTLV	Citrus tatter leaf virus isolate TL101		6494
MT720885		CiVA	Citrus virus A RNA1 isolate 1.8		6690
MT720886		CiVA	Citrus virus A RNA2 isolate 1.8		2731
KY110716.1		HSVd	Hop stunt viroid strain R140902-7		302
KY110717.1		HSVd	Hop stunt viroid strain R120621-2 (Cachexia)		295
KY110718.1		CDVd	Citrus dwarfing viroid strain R140910-12		297
KY110721.1		CEVd	Citrus exocortis viroid strain R140902-18		372

The average distance between the paired-end Illumina reads was calculated by mapping the paired-end data to the *C. sinensis* chloroplast genome (DQ864733) using CGW (default parameters).

### Read count normalisation

The read count of each reference gene, virus and viroid sequence was normalised for comparisons by calculating the transcripts per million (TPM) count. Firstly, the read count per kilobase of sequence (RPK) was calculated for each gene, virus and viroid sequence. After which the sum of all the RPKs were divided by a million to calculate the denominator for the transcripts per million (TPM) count for reference genes and pathogens separately. Each sequence’s RPK was divided by the reference genes or pathogens specific denominator to normalise the read count for biological and technical variation between samples. The TPM count for each reference sequence was used to compare the proportion of reads that mapped to a specific reference in each sample. Statistical analyses were performed using R available from the Comprehensive R Archive Network (CRAN) [54]. The Kruskal–Wallis rank sum test was used to compare data distributions [55]. Principal component analysis were visualised using package ggbiplot [56].

### Sequencing depth simulation

To assess the influence of data set size on the detection of virus and viroids using read mapping, data for each sample were sub-sampled randomly 10 times for 9 different subset sizes (1000, 5000, 10,000, 50,000, 100,000, 500,000, 1,000,000, 5,000,000, 10,000,000 reads) using seqtk (https://github.com/lh3/seqtk). The Illumina subsets were created in paired-end mode to have a total read count for each subset equal to the selected subset sizes (e.g. the 1000 reads subset from Illumina data contain 500 read pairs). The data subsets were mapped to the different reference genes or pathogen sequences using the abovementioned pipeline.

## Results

### Plant material

The plant material selected for HTS analysis was screened with RT-PCR and PCR to determine the infection status of the plants. The transmission of CLaf was unsuccessful and none of the plants selected for further analyses had a detectable CLaf infection. The negative control plant tested negative for all the pathogens tested for and the three infected plants tested positive for HSVd, CDVd, CEVd, CTV, CiVA and CTLV. The RT-PCRs to determine the CTV genotype status of the individual biological replicates confirmed the presence of CTV genotypes RB, VT, T3, T30 and S1.

The average RIN value for the RNA extracted for the HTS analysis with the CTAB method was 8.1 (± 0.1 standard deviation) and the average ribosomal RNA (rRNA) ratio was 5.5 (± 1.3 standard deviation). The average RIN value for the RNA extracted with the Zymo Research kit was 7.9 (± 0.5 standard deviation) and the average ribosomal RNA (rRNA) ratio was 1.9 (± 0.3 standard deviation. The Zymo Research kit extracted RNA was flagged by Macrogen due to an abnormal 5S peak.

### Data quality

On average 26 million paired-end reads were received per sample from the Illumina HTS. The quality of the data was high and after stringent quality trimming 96% of the data per sample was retained (Additional file 2). The average read length per sample was 99 nucleotides (nt) and the average read distance between the 5′ ends of the paired end reads was 196 bases. An average of 4.7 giga bases of Illumina data was obtained per sample. The Ion Torrent HTS yielded an average of 40 million reads per sample (Additional file 2) with an average read length of 137 nt. An average of 5.6 giga bases of Ion Torrent data was obtained per sample.

### De novo assembly

The Illumina data were de novo assembled using SPAdes into an average of 72,000 (CTAB extractions) and 79,000 (Zymo Research kit extractions) scaffolds with an average N50 of 1800 and 1600 nt, respectively (Additional file 2). The Ion Torrent data assembled into an average of 60,000 (CTAB RNA) and 18,000 (Zymo Research kit) scaffolds with an average N50 of 1300 and 1400 nt, respectively (Additional file 2).

The nontarget positive control *Ornithogalum* data assembled into 182,778 scaffolds with an N50 of 1219. No citrus pathogens were identified in either the *Ornithogalum* data or the *C. sinensis* negative control sample data (Additional file 2). Ornithogalum mosaic virus (OrMV) and ornithogalum virus 3 (OV3) scaffolds were identified in the *Ornithogalum* sample (Additional file 2). Conversely, no OrMV or OV3 scaffolds were detected in the any of the *C. sinensis* samples (Additional file 2).

**Fig. 2:**
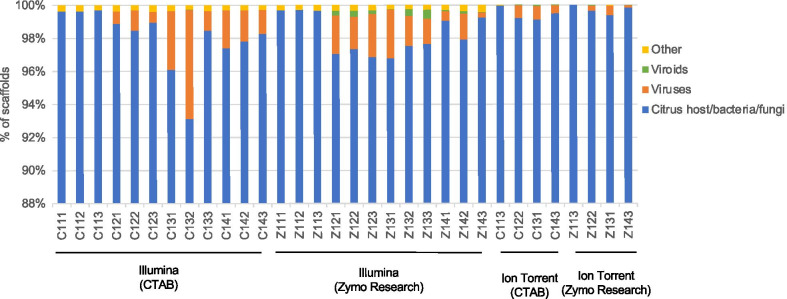
Stacked column chart displaying the ratio of identified SPAdes scaffolds per sample. The percentage of scaffolds per sample identified using Blastn classified into four categories i.e. other (unidentified), viroids, viruses and *Citrus sinensis* host, bacteria and fungi is displayed

On average 98.4% of the SPAdes assembled scaffolds from the *C. sinensis* samples were identified as host plant, bacterial or fungal sequences (Fig. 2). On average, 1.2% of the scaffolds were identified as virus sequences and 0.07% as viroid scaffolds (Fig. 2) and 0.2% of the scaffolds could not be identified. More viroid scaffolds were identified per sample from the Illumina data generated from the Zymo Research kit extracted RNA (0.23%) compared to the data from the CTAB extractions (0.01%) (Fig. 2).

Blastn analyses of the Illumina data showed the complete viral and viroid profiles expected for all the biological replicates (Additional file 2). However, no scaffolds with identity to viroid species CDVd (sample C122) and HSVd (sample C133), were assembled in one of the three technical replicates for two different biological replicates (both CTAB extractions). The de novo assembly of the Ion Torrent data performed less optimally with none of the 6 infected technical replicates containing scaffolds for the full pathogen complement (Additional file 2). No additional virus and viroid sequences were identified in any of the samples.

The Illumina data was assembled using CGW into an average of 66,315 contigs with an average N50 of 846 while the Ion Torrent data assembled into an average 12,347 contigs with an average N50 of 377. Analyses of the Blastn results for the Illumina assembled contigs showed that only nine samples showed the complete viral and viroid profile. No contigs with identity to HSVd were assembled from the four CTAB extracted samples (representing two biological replicates) and five of the Zymo Research kit extracted samples (representing three biological samples). Contigs could not be assembled from the Ion Torrent data for CTLV in one CTAB extracted sample, HSVd in three samples (one CTAB and two Zymo Research kit extractions), CDVd in one CTAB extracted sample and CEVd in two samples (one per extraction method) (Additional file 2).

Viroid results were inconsistent irrespective of sequencing platform or de novo assembler used. Neither assembler yielded scaffolds/contigs for HSVd in sample C133 (Illumina), CTLV in sample Z122 (Ion Torrent), CEVd in sample Z131 (Ion Torrent) and HSVd and CEVd in sample Z143 (Ion Torrent).

### *C. sinensis* reference gene read mapping

**Fig. 3:**
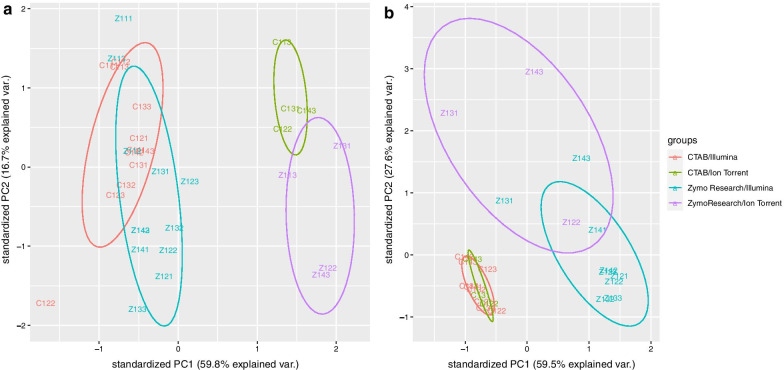
Principal component analysis (PCA) plot of the transcript per million (TPM) counts. The trends exhibited by the expression profiles of the RNA extraction and sequencing platform protocols (CTAB/Illumina, Zymo Research/Illumina, CTAB/Ion Torrent, Zymo Research/Ion Torrent) are displayed for the reference gene TPM counts in **a** and for the pathogen TPM counts in **b**. Samples are indicated with colours correlating to extraction method and sequencing platform and each colour circle represents a cluster of these designations

Reads were mapped to 12 *C. sinensis* reference genes to identify biological variation and technical variation associated with RNA extraction method and sequencing platform (Table 1). For comparison of the proportion of reads that mapped to a gene in each sample the TPM values were used. A principal component analysis of the gene data showed clear data point clusters indicating the variation between the different RNA extraction and sequencing platform protocols (Fig. 3A).

**Fig. 4:**
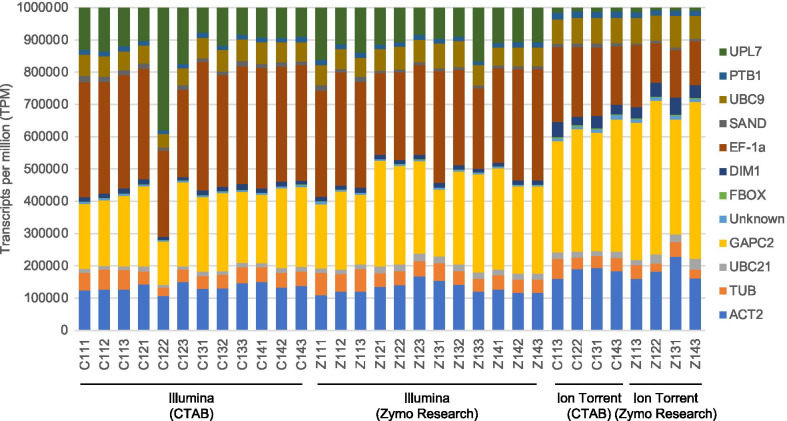
Stacked column chart displaying the transcript per million (TPM) values for each reference gene analysed. The ratios of the TPM count of each gene relative to the TPM count of the other genes are displayed for each sample subjected to the four RNA extraction and sequencing platform protocols (CTAB/Illumina, Zymo Research/Illumina, CTAB/Ion Torrent, Zymo Research/Ion Torrent)

The genes with the highest TPM count across all samples were GAPC2, EF-1a and ACT2 (Fig. 4). The genes with the lowest TPM count across all samples were FBOX and the unknown gene (Fig. 4). On average the highest coefficient of variance were observed with the CTAB extractions and the Illumina sequencing platform (Additional file 3). The ratio of each reference gene’s TPM count shows a consistent pattern per sequencing platform (Fig. 4). The gene expression based on the relative TPM counts showed significant difference (p-value < 0.05) between platforms across and between extraction methods for 11 and 10 of the genes, respectively (Fig. 4, Additional file 4). The most variation in TPM count between platforms is observed for UPL7 which has a significant lower TPM count compared to the other genes in the Ion Torrent data for both extraction methods (p-value < 0.05) (Additional file 4). GAPC2 also has a higher TPM count in the Ion Torrent data compared to the Illumina data (p-value < 0.05) (Additional file 4). The relative ratio of EF-1a is higher in the Illumina data compared to the Ion Torrent data (p-value < 0.05) (Additional file 4).

**Fig. 5:**
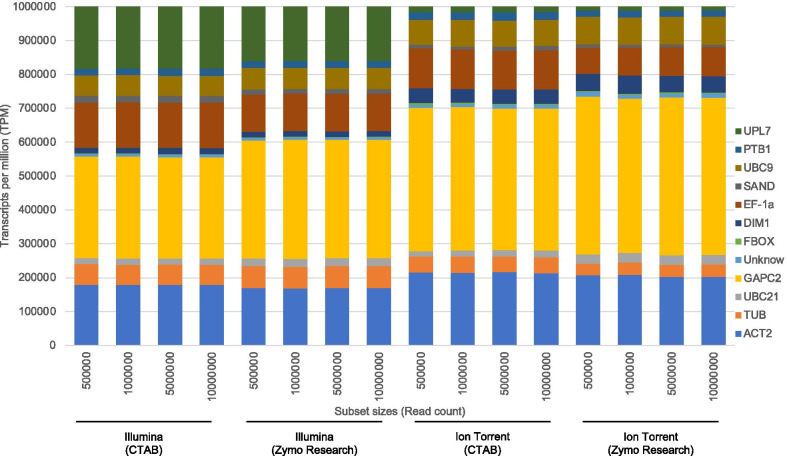
Stacked column chart displaying the average transcript per million (TPM) values for each gene transcript. The average ratios of the TPM count of each *Citrus sinensis* gene relative to the TPM count of the other genes are displayed for four different subset sizes (500,000, 1,000,000, 5,000,000, 10,000,000) for each of the RNA extraction and sequencing platform protocols (CTAB/Illumina, Zymo Research/Illumina, CTAB/Ion Torrent, Zymo Research/Ion Torrent). The average TPM per gene for 10 replicates of each subset is displayed

The comparison between extraction methods showed no significant differences between the ratios of the gene TPM counts for the biological/technical replicates (Fig. 4). Only three of the genes showed significant differential expression between extraction methods independent of sequencing platform (p-value < 0.05) (Additional file 4), while five genes showed significant differential expression between extraction methods for the Illumina sequencing platform and only three genes for the Ion Torrent sequencing platform (p-value < 0.05) (Additional file 4). The relative ratio between the 12 different genes’ TPM counts remained consistent across the different subset sizes (Fig. 5). The same variation in the TPM count of UPL7 and GAPC2 between the two sequencing platforms was observed across different subset sizes.

### Pathogen read mapping

Reads were mapped to reference genomes of the viruses and viroids identified using the expected virome including CTV genotype RB, CiVA RNA 1 and 2, CTLV, HSVd, CDVd and CEVd (Table 1). Concurrently, reads were also mapped to the nontarget CTV genotype T68 and the Cachexia causing variant of HSVd [57, 58]. The principal component analysis of the pathogen TPM count data showed clear clusters of data points indicating the variation between the different extraction/platform protocols (Fig. 3b).

**Fig. 6:**
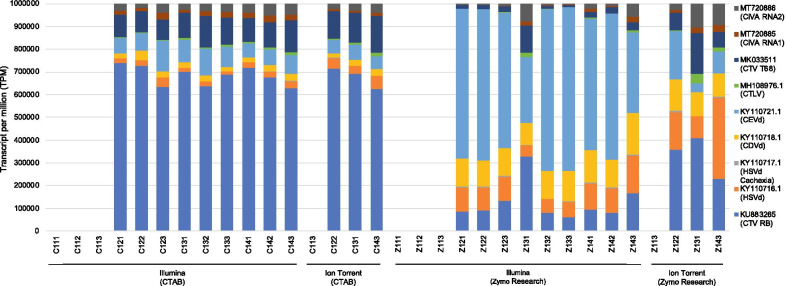
Stacked column chart displaying the transcript per million (TPM) ratio for each pathogen reference analysed. The ratios of the TPM count of each pathogen relative to the TPM count of the other pathogens are displayed for each sample subjected to the four RNA extraction and sequencing platform protocols (CTAB/Illumina, Zymo Research/Illumina, CTAB/Ion Torrent, Zymo Research/Ion Torrent)

The expected virome was detected using read mapping with all four extraction/platform protocols. The TPM count for each pathogen for each extraction/platform protocol is illustrated in Fig. 6. The viroid TPM component of the CTAB extracted samples were much smaller compared to the Zymo Research kit extracted RNA samples, independent of sequencing platform. More variation in pathogen TPM count between the technical replicates of each biological sample was also observed in the Zymo Research data (Fig. 6).

**Fig. 7:**
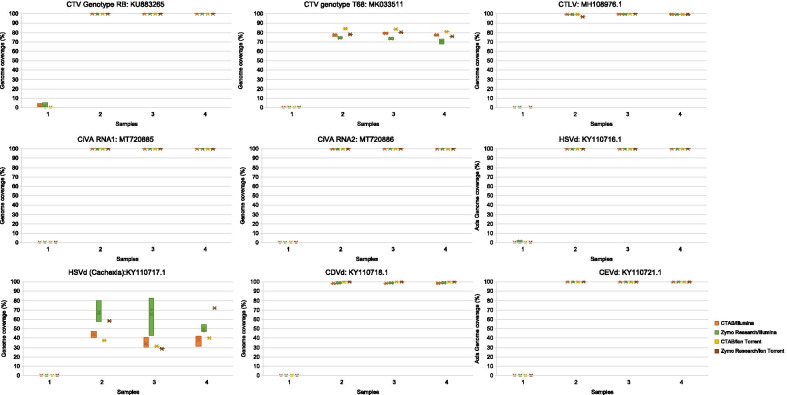
Box and whisker plots of the genome coverage of each virus and viroid reference sequence. The plot illustrate the variation in virus and viroid genome coverage (percentage of bases of the reference genome covered) after read mapping per RNA extraction and sequencing platform protocol (CTAB/Illumina, Zymo Research/Illumina, CTAB/Ion Torrent, Zymo Research/Ion Torrent) for each biological replicate. Sample 1: Healthy, Sample 2-4: Infected

All protocols obtained more than 99% genome coverage (percentage of bases covered on the reference genome) for CTV genotype RB (KU883265), CTLV (MH108976.1), HSVd (KY110716.1), CEVd (KY110721.1), and CiVA RNA1 (MT720885) and CiVA RNA2 (MT720886) (Fig. 7). The only exception was CDVd (KY110718.1) that had a genome coverage of 100% with the Ion Torrent protocol irrespective of extraction method. With the Illumina protocol an average of 98.4% and 99% coverage was obtain with CTAB extractions and the Zymo Research kit, respectively (Fig. 7). Read mapping to the nontarget CTV genotype T68 showed an average genome coverage of only 78%, 73%, 83% and 78% for the CTAB/Illumina, Zymo Research/Illumina, CTAB/Ion Torrent and Zymo Research/Ion Torrent protocols, respectively. Read mapping to the nontarget Cachexia causing variant of HSVd showed only 38%, 61%, 36% and 53% average genome coverage for the four extraction/platform protocols (Fig. 7).

The *Ornithogalum* nontarget positive control data was also mapped to all the citrus pathogens to evaluate the level of cross-contamination between samples. Only seven, two and one reads mapped to CTV (KU883265), CTV (MK033511) and CiVA (MT720885), respectively. The citrus Illumina data sets were also mapped to OrMV (KY769694.1) and on average 197 reads (minimum:32, maximum:788) mapped from the CTAB/Illumina data and 60 reads (minimum:0, maximum:182) of the Zymo Research/Illumina data mapped. The negative control data was also mapped to all the pathogens and the highest read count obtained was 22 reads (Illumina) and one read (Ion Torrent) for CTV (KU883265).

### Sequencing depth

**Fig. 8:**
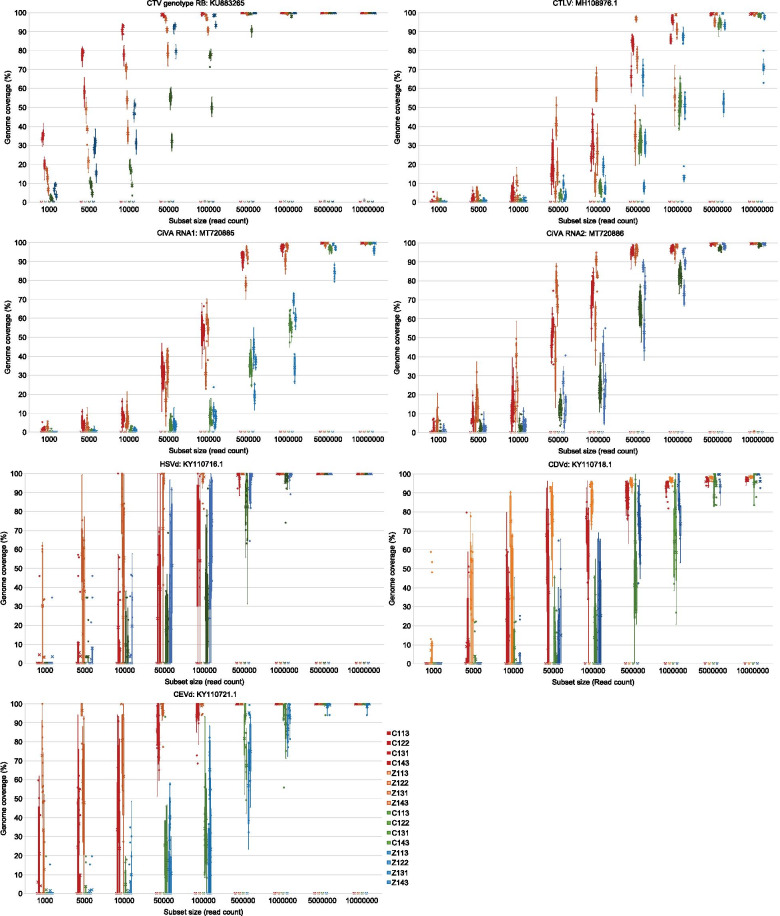
Box and whisker plots of pathogen reference genome coverage. The variation in genome coverage (percentage of bases of the reference genome covered) at different sequencing depths is illustrated by mapping each subset of reads to the different pathogen accessions. Only the technical replicate of the sample that was sequenced on both platforms is displayed. Each data set size was randomly selected 10 times from each sample. The RNA extraction and sequencing platform protocols is shown with different colours (Red: CTAB/Illumina, Orange: Zymo Research/Illumina, Green: CTAB Ion/Torrent, Blue: Zymo Research/Ion Torrent)

**Fig. 9:**
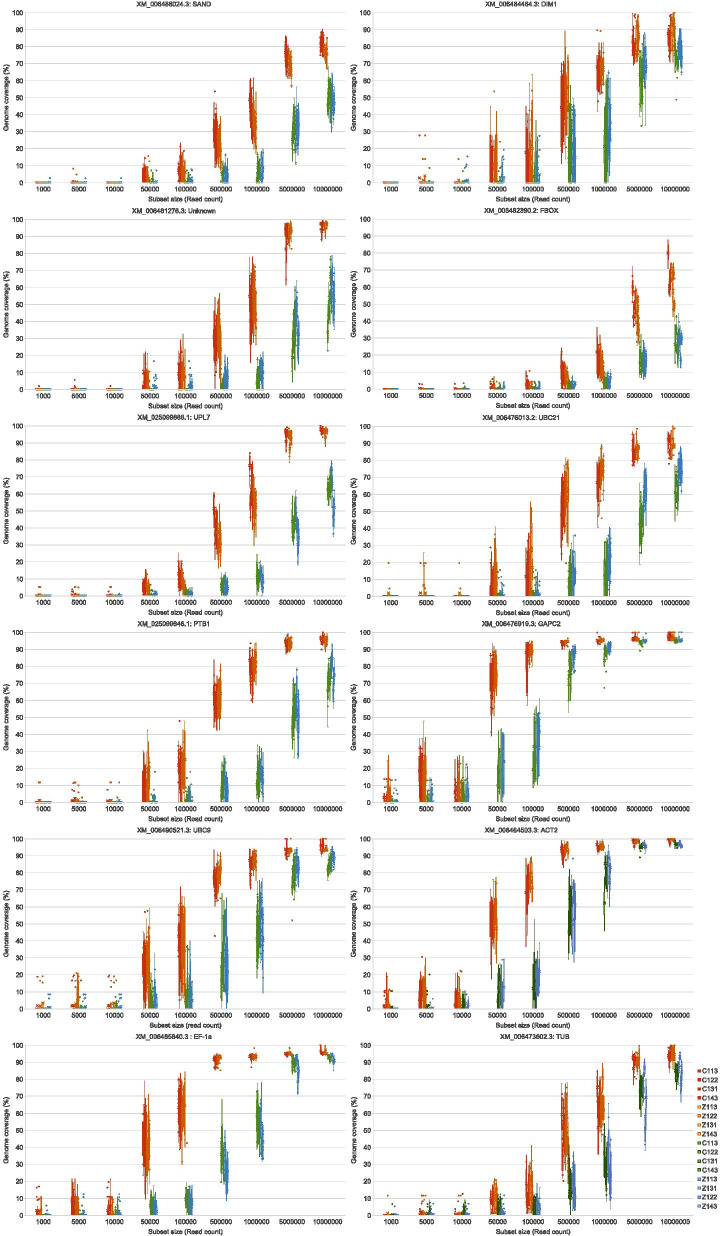
Box and whisker plots of reference gene coverage. The variation in genome coverage (percentage of bases of the reference genome covered) at different sequencing depths is illustrated by mapping each subset of reads to the different gene accessions. Only the technical replicate of the sample that was sequenced on both platforms is displayed. Each data set size was randomly selected 10 times from each sample. The RNA extraction and sequencing platform protocol is shown with different colours (Red: CTAB/Illumina, Orange: Zymo Research/Illumina, Green: CTAB/Ion Torrent, Blue: Zymo Research/Ion Torrent)

Subsampling the original data into nine different sized data subsets showed the influence of sequencing depth on the potential genome/transcript coverage that can be obtained. Overall, the Illumina data reached a higher genome coverage with less data for both the pathogen and gene reference sequences (Figs. 8 and 9).

With the 10,000,000 reads subset an average target pathogen genome coverage of 99.5%, 99.6%, 99.2% and 98.0% was obtained for the CTAB/Illumina, Zymo Research/Illumina, CTAB Ion/Torrent and Zymo Research/Ion Torrent protocols, respectively (Additional file 5).

Since a previous CTV study showed that it is possible to obtain up to 90% genome coverage for nontarget genotypes [46], a 90% genome coverage threshold was selected to evaluate for each of the virus and viroid accessions. Even though it was possible to obtain more than 90% genome coverage for CTV with the 50,000 reads subset (all subset replicates from each technical replicate) using the CTAB/Illumina protocol, the one million reads subset was required to obtain at least 90% coverage for CTV with all four extraction/platform protocols (Additional file 5, Fig. 8).

To obtain a more than 90% genome coverage for all target pathogens the five million reads subset was needed with the CTAB/Illumina protocol. However for the other three extraction/platform protocols at least the ten million reads subset was required for more than 90% genome coverage consistently over all subset and technical replicates (Additional file 5).

The five million reads subset of the CTAB/Illumina protocol was able to consistently obtain a more than 90% genome coverage for all viruses and viroids, separately. However, with the Zymo Research/Illumina protocol at least 90% genome coverage of the viruses was obtained with the ten million reads subset and for the viroids with the one million reads subset (Additional file 5). The CTAB/Ion Torrent protocol required the ten million and five million reads subset for at least 90% genome coverage of the viruses and viroids, respectively while the Zymo Research/Ion Torrent protocol required the ten million reads subset for both viruses and viroids (Additional file 5).

The Zymo Research/Ion Torrent protocol data was unable to obtain a 90% or higher CTLV genome coverage consistently for all replicates (Additional file 5). The most variation in genome coverage at the lower data set sizes was obtained for the viroid sequences (Fig. 8).

The average reference gene accession coverage with the 10,000,000 reads subset size was 91.3%, 89.7%, 71.3% and 73.4% for the CTAB/Illumina, Zymo Research/Illumina, CTAB/Ion Torrent and Zymo Research/Ion Torrent protocols, respectively (Additional file 5).

## Discussion

In this study an experimentally constructed citrus virome was characterised using HTS to evaluate the influence of sampling, RNA extraction method, sequencing platform and data analysis pipeline. Four sweet orange (cv. ‘Madam Vinous’) trees were prepared, one negative control and three graft inoculated with CTV, CTLV, CiVA, HSVd, CDVd and CEVd. Each plant was sampled three times at the same timepoint, and RNA extracted using two different methods. Two sequencing platforms were selected to generate data from three samples from four plants (Illumina) and one sample from four plants (Ion Torrent). All data sets were subjected to a reference independent de novo assembly approach and a dependent read mapping strategy to determine the virome profile of each plant sample.

HTS can be utilised for both detection and discovery and the acceptable level of variation in specificity, sensitivity and repeatability that can be tolerated will depend on the application. In the present study, the detection of virus and viroid species were evaluated for application as a routine diagnostic assay. Identifying the exact virus or viroid variant, for example, was therefore not required for pathogen detection.

HTS is intrinsically specific and is therefore only limited by the accuracy of the base calls, the depth of the data and the completeness of the reference databases [15]. A public reference database can be incomplete due to novel viruses or new variants of viruses that are yet to be discovered. Local databases require continuous upkeep to be complete, even if it is only for virus species or variant additions. Nonetheless, novel pathogens or different variants of known pathogens not contained in a local database can, in some cases, still be detected by de novo assembly followed by homology searches and read mapping, just with lower confidence levels and less robustness. The limitations of such databases should be considered throughout the data analysis and interpretation.

The composition of the starting nucleic acids additionally impacts detection specificity. The virome profiles of the Zymo Research kit extracted samples were less consistent across samples and replicates than the profiles of the CTAB extracted samples (Fig. 6). This variation can be due to several fundamental differences in the RNA extraction protocols. The input plant material amount for the CTAB extraction method is one gram compared to the 200 mg input for the Zymo Research kit. It is more difficult to obtain a representative sample using a lower input extraction method. This can potentially lead to the generation of false negative results if the virome includes pathogens that are unevenly distributed in the plant as was observed for plum pox virus (PPV) [59] and certain grapevine viruses [60].

The lower weight input restriction of the Zymo Research RNA extraction kit resulted in greater variation in the virome profiles between the technical replicates of each biological sample (Fig. 6), probably due to sectorial differences in concentrations of the target pathogens in the plants. The CTAB method yielded more consistent virome profiles between samples, likely due to the ability to process a more homogeneous sample, however this method appears to have a slight bias against viroid sequences. Collectively, this virome analysis indicates that a low weight input extraction method has risk implications for use in a routine HTS detection assay.

Reproducibility is required at each step of the HTS assay, from nucleic acid extraction to data interpretation. Previous studies highlighted the link between appropriate depth of coverage and the repeatability of the assay [37, 40], but no systematic studies on repeatability and reproducibility have been published. In this study an attempt at reproducibility was made by including both biological and technical replicates for the Illumina sequencing protocol.

The two different extraction methods yielded total RNA with different rRNA profiles. No significant difference between the RIN values for the two groups was observed, however the rRNA ratio of the Zymo Research kit extracted RNA was significantly lower than for the CTAB RNA due to a higher concentration of 5S rRNA yielded by the Zymo Research kit. This indicates a potential difference in the RNA species extracted with each method and it is possible that the CTAB extraction selected against viroid sequences due to the Lithium Chloride (LiCl) precipitation step. The SPAdes de novo assembly of the Illumina data did not produce viroid scaffolds for one technical replicate of two different CTAB extracted samples, compared to all the expected viroid scaffolds assembled in the Zymo Research kit replicates (Additional file 2). The SPAdes assemblies of the Zymo Research data also generated more viroid scaffolds compared to the CTAB data (Fig. 2). The read mapping strategy also displayed the difference in viroid RNA concentration between the two extraction methods where the ratios of pathogen concentration was vastly different between the extraction methods, independent of the sequencing platform (Fig. 6). More viroid RNA reads were obtained using the Zymo Research kit. Due to the potential lower representation of viroid RNA obtained with certain extraction methods and the small genome size of viroids, it is possible that a viroid infection may be missed with only a de novo assembly approach. Although, the RNA extraction method influenced the performance of the detection assay in the present study, it did not alter the final combined de novo and read mapping results (Additional file 2, Additional file 5) and all pathogens were consistently detected.

The selection in de novo assembler can influence the result as seen in the contigs assembled with CGW compared to the SPAdes scaffolds (Additional file 2). The SPAdes assembly with the Illumina data performed better in confirming the expected virome profile compared to the assemblies with the Ion Torrent data. Both assemblers were able to assemble more and longer contigs/scaffolds with the Illumina data. Even though 1.2 times more Ion Torrent data than Illumina data was obtained from the service providers, the 196 nt read distance between the paired-end reads of the Illumina data may contribute to better contig assemblies compared to the single-end Ion Torrent reads with an average length of 137 nt.

The principal component analyses using the TPM counts of the pathogen and gene accession read mappings showed a clear separation between the different extraction/platform protocols (Fig. 3). However, for the gene accessions, the most variation was between sequencing platforms and for the pathogens the variation was between RNA extraction methods (Fig. 3). This is partially explained by the viroid component of the pathogen profile that was greater in the Zymo Research data sets. The variation between technical replicates was however minimal and the variation observed was rather as a result of extraction method or sequencing platform.

The investigation into the expression profile of reference genes allowed the comparison between samples across different extraction/platform protocols to potentially answer questions relating to the suitability of the sequencing depth to address pathogen detection. The expression pattern of these genes is hypothesized to be stable and even if the gene expression is modulated in response to biotic stress, the variation between samples should be reflected in each of the different extraction/platform protocols selected. By identifying low and high expressing genes, gene expression profiles can be used as internal controls for RNA extraction efficiency, library construction and also to determine the number of reads required for accurate detection. Using the host reference gene mapping ratios, outlier samples can be identified as seen for sample C122 (Illumina) (Fig. 4). The expression pattern of the 12 genes selected in this study showed a consistent pattern across extraction methods but differed for each of the sequencing platforms (Fig. 4). No significant variation in expression patterns were observed between healthy and infected plants. The different pattern per sequencing platform was also consistent, independent of data set size (Fig. 5). Therefore, based on the data generated in this study, UPL7 and GAPC2 might not be consistent internal controls for cross platform comparisons, however, when selecting a single platform, these genes can be used. The reference genes can also be used to normalise the virus or viroid TPM count to allow for direct virus and viroid concentration comparisons between samples. Only five of the gene accessions had a consistent gene coverage of more than 90% for all technical and subset replicates (Additional file 5).

The expected virome was confirmed with RT-PCR and included five CTV genotypes (RB, VT, T3, T30 and S1), CTLV, CiVA and three viroids (HSVd, CDVd and CEVd). The influence of read mapping to a distant variant of the target virus/viroid was assessed by including nontarget reference sequences of CTV (genotype T68) and the Cachexia variant of HSVd. An average genome coverage for CTV genotype T68 of 73–83% was obtained for the four different extraction/platform protocols. Compared to the genome coverage of the expected CTV genotype RB of > 99%, the T68 read mappings are distinguishable as false positive mappings. A previous study showed that it is possible to obtain up to 90% coverage for nontarget genotypes in mixed genotype infections and that read mapping across more than 95% of the genome is indicative of the presence of a particular genotype [46]. Due to the extent of variation between CTV genotypes (2–9%) [46], the selection of reference sequences will influence the coverage percentage. Therefore, if a reference for a genotype not present in the data is used for read mapping, a lower percentage would still be indicative of the presence of CTV, but just that a different CTV genotype than the reference would be expected. This would be true for most viruses and by including representative sequences of the different genotypes in the read mapping strategy, false negative diagnostic calls can be prevented. In the case of HSVd it is important to be able to differentiate between disease causing and latent variants since they are biologically distinct in citrus. The nontarget Cachexia variant of the HSVd genome was only 36%-61% covered for the four different extraction/platform protocols, clearly indicating that this variant was not present in the samples.

The sensitivity of any detection assay is directly linked to the proportion of viral RNAs among the host cellular RNAs. Therefore, sequencing depth plays an important role in the reliability of the HTS assay. The main conclusion from the subset experiment in this study was that less Illumina data was needed to obtain complete or near complete genomes of the expected pathogens and the Ion Torrent data can perform on par with Illumina if more reads are used for the read mapping. The number of bases in each subset size for the Ion Torrent data was 1.3–1.4 times more compared to the Illumina subsets as a consequence of the longer read length. However, the average distance of 196 bases between the Illumina read pairs may have increased the efficacy of the Illumina read mapping.

The finding of a previous study [37] that showed that sequencing one million reads will provide sufficient genome coverage for closterovirus detection, was confirmed (Fig. 8, Additional file 5). It was also shown that a higher number of reads is needed for other pathogens depending on the extraction/platform protocol. Viroid detection was shown to be variable and even though it was possible to obtain a complete genome with lower read numbers, the detection is only consistent with more sequencing depth.

No citrus pathogen sequences were de novo assembled from the *Ornithogalum* nontarget positive control or *C. sinensis* negative control data that was included in the Illumina and Ion Torrent sequencing runs and no pathogens associated with *Ornithogalum* were assembled from the citrus RNA data. The *Ornithogalum* data set were mapped to the citrus pathogens and negligible read counts were obtained. A maximum of 788 citrus RNA reads from the different samples mapped to ornithogalum mosaic virus however the genome coverage never reached more than 1.5%. This indicated no significant cross-contamination between samples.

The reproducibility of this study was not measured specifically, as a true test of reproducibility would require an interrogation of the extent to which consistent results could be obtained by repetition of the whole experiment at different timepoints. An attempt at reproducibility was made to include both biological and technical replicates for the Illumina sequencing protocol. The technical replicates were however not sequenced on the Ion Torrent platform due to a cost implication. The Ion torrent data cost 43% more for the same amount of Illumina data.

In this biological context, reproducibility will not be completely achievable as variables such as plant age, growth stage and virus concentration linked to infection duration might influence the outcome.

A comparison of single-end data from the Illumina platform and single-end data from the Ion Torrent platform was not evaluated to keep the generation of data as close to a real-case scenario as possible. The two service providers, Macrogen and CAF, provide by default, paired-end Illumina and single-end Ion Torrent data, respectively.

## Conclusions

This study is a detailed measurement of technical variation in HTS data associated with the detection of viruses and viroids in citrus. The study evaluated the efficiency of using HTS to detect two single stranded RNA viruses from different families, a negative-sense single-stranded RNA virus and three viroid species. The study evaluated the influence of RNA extraction protocol, sequencing platform and data analysis pipelines on the sensitivity, specificity and repeatability of HTS as a detection tool. Each of these parameters introduce a different bias that creates variation in the data output. Even though the different extraction methods, sequencing platform and data analysis tools resulted in variation in the present study, the end result being the virome profile of each sample could be confirmed independent of the HTS approach. The study highlights the need to be aware of the level of variation associated with each approach in strategy, from sample collection to data interpretation and how these variables may impact on the initial objective of the HTS assay. This awareness is critical to enable informed adjustments to correctly interpret the data for a reliable results. The primary recommendation that follows from this study is that, irrespective of extraction method or sequencing platform, a combination of de novo assembly and read mapping be used for a routine detection assay.

Since the goal of this study was to evaluate HTS as a detection tool in quarantine or certification schemes, and not for discovery purposes, a list of known pathogens should be available in these settings for read mapping. The aim of a de novo assembly in the certification scheme context will be to identify unsuspected pathogens.^1^ The absence of virus/viroid related de novo assembled contigs does not automatically indicate a negative status for the respective pathogen and read mapping is required as a validation step to confirm absence. This is especially necessary for low concentration viruses and viroids. Read mapping against multiple reference genes as internal controls is also recommended to establish gene ratios for a specific assay. This allows for the evaluation of sequencing depth to accurately determine the absence of low-level infections. The inclusion of a nontarget positive and a negative control can assist significantly to evaluate cross contamination between samples. The final conclusion is that sequencing depth matters and that with enough data the variation observed between extraction methods and sequencing platforms are diminished and equivalent results can be obtained.

The application of HTS for the detection of plant viruses is commonly described as being unbiased, however this is only true within a specific context, in that it does not require any prior knowledge of the pathogens. There are however, slight biases and variations at every step of an HTS assay, as demonstrated, but which can easily be corrected for when quantified. This study provides strong evidence that the application of HTS for routine pathogen detection is attainable if the detection pipeline is critically validated.

## Supplementary Information

**Additional file 1**Species and strain-specific primer sequences used in a two-step RT-PCR to amplify citrus viruses and viroids.

**Additional file 2**Descriptive statistics of high-throughput sequencing data generated and the identification of contigs/scaffolds per* de novo* assembly.

**Additional file 3**Descriptive statistics of the TPM values for the different gene accessions used as internal control references.

**Additional file 4**Statistical tests performed using the Kruskal-Wallis rank sum test between the data distributions of the different extraction methods and sequencing platform data sets based on transcripts per million (TPM) counts per gene analysed. Significant differences are indicated in bold if the p value < 0.05.

**Additional file 5**The subset sizes for which the respective accession obtained a 90% and 95% genome coverage for all sample and subset replicates with read mapping per RNA extraction method and sequencing platform protocol.

## Data Availability

The datasets used and/or analysed during the current study are available from the corresponding author on reasonable request.

## Abbreviations

HTS: High-throughput sequencing
CTV: Citrus tristeza virus
CTLV: Citrus tatter leaf virus
HSVd: Hop stunt viroid
CDVd: Citrus dwarfing viroid
CEVd: Citrus exocortis viroid
CiVA: Citrus virus A
CLaf: Candidatus Liberibacter africanus
CRI: Citrus Research International
cDNA: Complementary DNA
dNTPs: Deoxyribonucleotide triphosphate
OrMV: Ornithogalum mosaic virus
RIN: RNA integrity number
bp: Base pair
nt: Nucleotide
CGW: CLC genomics Workbench
BWA: Burrows–Wheeler Alignment Tool
NCBI: National Center for Biotechnology Information
TPM: Transcripts per million
RPK: Read count per kilobase of sequence
CRAN: Comprehensive R Archive Network
rRNA: Ribosomal RNA
CTAB: Cetyltrimethylammonium bromide
RNA: Ribonucleic acid
OV3: Ornithogalum virus 3
GAPC2: Glyceraldehyde-3-phosphate dehydrogenase C2
EF-1a: Elongation factor 1-alpha
ACT2: Actin-2
FBOX: F-box family protein
UPL7: Ubiquitin-protein ligase 7
cv.: Cultivar
sRNA: Small RNA
PPV: Plum pox virus
LiCl: Lithium chloride

## Footnotes

1 The primary application described here is for routine virus detection and hence the de novo assembly step is not critical for this application. However, it would be short-sighted not to include this step that provides an extra layer of security. Unsuspected or novel viruses detected in these assays will require additional validation, contrary to the detection of known pathogens for which this assay was validated.
